# Revisiting L2 pragmatic competence through implicit vs. explicit instructional framework

**DOI:** 10.3389/fpsyg.2022.987729

**Published:** 2022-08-22

**Authors:** Nan Huang

**Affiliations:** School of Foreign Languages, Xinyang College, Xinyang, China

**Keywords:** pragmatic competence, real communication, authentic social setting, explicit instruction, implicit instruction

## Abstract

Successful interaction in the target language requires L2 learners to use and understand the grammatically correct language. At the same time, the language used is expected to produce socioculturally appropriate utterances that it refers to their Pragmatic competence. The latter entails acquiring pragmatic competence, which has proved to be very challenging for L2 learners. This is because they gain limited exposure to the use of language for real communication in an authentic social setting. Moreover, instruction has been found to influence the functional abilities in L2 as it equips the learners with the ability to produce and comprehend L2 in different situations. Focusing on the research conducted on the role of explicit and implicit instruction on L2 pragmatic competence, this study aimed to give a summative description of the empirical studies carried out on teaching pragmatics. The investigation ends up with a conclusion, instructional implications, and suggestions for future research.

## Introduction

As a central component of communicative competence, pragmatic competence takes in the capability to engage in the dynamic and interactive negotiation of meaning. Such a type of negotiation takes place between two or more persons in specific circumstances (Timpe Laughlin et al., 2015). Similarly, LoCastro (2003) asserts that pragmatics concerns the examination of the meaning conveyed between speaker and hearer thanks to their joint actions, which involves both linguistic and non-linguistic cues embedded within socioculturally organized activities. This delineation of pragmatics emphasizes speaker-hearer communication unfolding in a sociocultural setting; therefore, it can be implied that pragmatic competence requires knowledge of linguistics, and social norms, as well as the capability to put to practice these forms of knowledge in social interactions. Pragmatic competence concerns the individual’s capability of understanding and making appropriate responses to communicative and linguistic gestures (Farashaiyan and Muthusamy, 2016). Both grammatical knowledge and pragmatic competence enjoy the same status in terms of the contribution they make to effective communication. As mentioned by Celce-Murcia (2007), the development of L2 proficiency entails the improvement of pragmatic competence. Lacking this ability, a speaker may grapple with miscommunication. Therefore, literature shows that in recent decades, there has been growing attention to the acquisition of pragmatic competence as an essential component other than lexico-grammatical proficiency in the EFL context (Ishihara and Cohen, 2010).

The integration of SLA and pragmatics yields L2 pragmatics. Studies on this construct examine the ways in which L2 learners gradually gain the knowledge of what, when, and to whom they should say something; what factors are contributing to gaining such knowledge; how can such knowledge be studied, taught, and assessed in social settings. These make up the field of L2 pragmatics (Bardovi-Harlig, 2013; Taguchi and Roever, 2017). From this perspective, L2 pragmatics is concerned with examining the association between L2 structure and its use by focusing on how these two abilities are learned and how they cooperate (Hymes, 1972). The results of investigations conducted in China reveal that EFL learners’ pragmatic competence is not satisfactory (Liu, 2004; Xiao et al., 2019). Although L2 learners perform well on a test, they grapple with miscommunication in English when they have to take part in authentic conversations. These challenges emanate from their poor pragmatic competence. EFL learners in China have rare opportunities for being exposed to real language use. Consequently, they acquire insufficient pragmatic knowledge, making it challenging for them to comprehend and produce language appropriately. Furthermore, classroom input makes an important contribution to English language learning (Kim and Hall, 2002), so they have proved to be indispensable elements in the progress of pragmatic competence (Bardovi-Harlig, 2013). As a result, it is essential to incorporate pragmatic instruction in language education programs.

Given that L2 learners need to be equipped with pragmatic competence to be able to engage in successful negations of meaning in the context of a target language, it is of enormous importance to incorporate effective education on pragmatic skills in language courses (Akutsu, 2012) as a review of the literature shows that an increasing number of investigations have been dealing with L2 pragmatics over the last 30 years (Taguchi and Roever, 2017; Derakhshan and Arabmofrad, 2018; Yang, 2018; Taguchi, 2019; Derakhshan and Eslami, 2020; Malmir and Derakhshan, 2020; Derakhshan and Shakki, 2021; Yang and Ke, 2021; Zhang, 2022). Yet, when it comes to pedagogical aspects, many L2 learning environments, teacher education programs, and textbooks fall short of providing L2 learners with pragmatic instruction and daily interaction skills and sub-skills (Bardovi-Harlig, 2013). Pragmatic knowledge used to be underestimated by many teachers and educators until recently, which is evidenced by the question posed by Kasper (1997) as to whether one can teach pragmatic competence in the classroom? Even though many objections and arguments have been made in this respect, much-supporting evidence has been collected to consolidate the claim that pragmatics is necessary and can be taught in an L2 setting.

Classroom observations show that L2 learners suffer from a deficiency in pragmatic knowledge, which emanates from exposure to insufficient and sometimes irrelevant input in EFL classrooms (Malmir and Derakhshan, 2020). There has been a controversy over whether or not pragmatics can lend itself to teaching. To this end, some investigations have examined the possible effect of explicit instruction of pragmatic skills and metalanguage on the quality of communication in the target language in classroom settings (Grice, 1975; Blight, 2002; Lee, 2002). The increasing number of studies on pragmatics has revealed that teachers can teach pragmatic competence in their classes (Yazdanfar and Bonyadi, 2016; Alzeebaree and Yavuz, 2017). Despite the arguments made by some linguists concerning the difficulty of teaching pragmatics (Krisnawati, 2011), it is highly important to integrate pragmatic knowledge in an L2 classroom. This is, particularly, essential for EFL contexts where instruction is the only means through which learners are exposed to the target language (Nugroho et al., 2020). In the same vein, in the view of Bardovi-Harlig (2013), teaching pragmatics can be useful as it paves the way for the L2 learners’ acquaintance with the second or foreign language by providing authentic materials. Therefore, students can experience the actual use of language. In the same vein, Kasper and Rose (2002) maintain that L2 learners would not acquire pragmatic competence by merely being exposed to the target language as they fail to become aware of many contextual factors, which are not noticed given that they are not salient in the context. Consequently, teaching pragmatic competence and socially appropriate language have been put forth as one way to overcome this shortcoming (Bardovi-Harlig and Mahan-Taylor, 2003).

Assumed the results of the formative paper presented by Norris and Ortega (2001) on the efficacy of L2 instruction, meta-analysis came to be prioritized over other research synthesis methods. Some investigations (Norris and Ortega, 2001; Badjadi, 2016; Plonsky and Zhuang, 2019; Yousefi and Nassaji, 2019; Shakki et al., 2021) have been carried out using a meta-analysis format and review studies to address the various topics in the area of pragmatics. Moreover, There has been a growing interest in examining the role of instruction on L2 pragmatics achievement in recent years (Rose, 2005), with the majority of them dealing with three significant questions: (1) can L2 pragmatic competence lend itself to teaching?; (2) can instruction make any contribution?; (3) does the type and method of instruction have any effect? In a nutshell, these studies have shown that although L2 learners find some areas of L2 pragmatics very difficult, L2 pragmatics lends itself to teaching, which has proved to contribute to pragmatic development (Kasper and Rose, 2002; Rose, 2005; Jeon and Kaya, 2006). Moreover, in the past, a large number of investigations have sought to demonstrate the efficacy of pragmatic instruction, with a growing body of research carrying out quasi-experiments in this regard (Taguchi, 2015). These studies have investigated pragmatics instruction by focusing on various issues, behaviors, and L2 structures. Some articles (Rose, 2005; Jeon and Kaya, 2006; Belz, 2007; Taguchi, 2015; Derakhshan et al., 2020), have presented a summary and meta-analysis of these research findings.

Overall, there is a consensus that instruction will turn out to be more effective than mere exposure to input; however, it should be stated that the effectiveness of L2 pragmatics instruction varies across studies. The foregoing studies have dealt with multiple factors assumed to contribute to the effectiveness of pragmatics instruction; however, very few studies have examined the contribution of different types of instructions in an EFL context. Considering the important contribution of pragmatic competence to L2 learning and the role it plays in allowing the learners to engage in effective communication in cross-cultural contexts, the present study sought to paint a general picture of the studies conducted on the instruction of L2 pragmatic competence. The aim was to provide the researchers with some insights on whether the instruction of pragmatic skills yields any positive outcomes in the EFL context.

## Review of the literature

### Pragmatic competence

Several descriptions of the term “pragmatics” have been presented in the literature, which addresses or highlights the various dimensions of the construct (LoCastro, 2003). For example, Thomas (1995) maintains that both speaker’s intention and statement play an important role in pragmatics, so the exclusion of one leads to the exclusion of the other one. LoCastro (2003) elaborates on the multiple characteristics of pragmatic competence, including its interactional and dynamic aspects. He defines pragmatic competence as the examination of the shared meaning created by speaker and hearer, which involves both linguistic and non-linguistic indicators in socioculturally prearranged tasks. This definition attaches great importance to the speaker–hearer communication, verbal and non-verbal channels (Beebe and Waring, 2004; Wharton, 2009), as well as the sociocultural factors determining the intended meaning in interactional discourse. According to Kasper and Rose (2002), pragmatic competence involves the capability of producing and comprehending statements or speech in sociocultural relations. Barron (2003) describes pragmatic competence as having at one’s disposal the linguistic resources required for comprehending specific illocutions. This requires knowing the progressive facets of speech acts, as well as how to use the particular language’s linguistic resources in appropriate contexts. Consequently, having pragmatic competence has to do with an interaction between linguistic knowledge and contextual use. This is because producing and comprehending words is highly dependent on grammatical knowledge and contexts.

Pragmatic competence involves the individual’s capability of dealing with different social situations through the use of language. According to Taguchi (2019), the conceptualization of pragmatic competence has changed over the years. This is because pragmatic competence has proved to be a multi-dimensional construct that entails three types of knowledge and skill: knowledge of what to say in a linguistically correct and socioculturally appropriate manner; the ability to engage in interaction to convey the message adaptively and flexibly in line with the changing contexts; and agency to figure out if it is helpful to put to use the knowledge in a given community.

One primary conceptualization of pragmatic competence was initially proposed by Leech (1983) and Thomas (1995). Some researchers have proposed their early conceptualizations of pragmatic competence, focusing on two sub-categories of pragmatics, namely, pragmalinguistics and sociopragmatics. The former is related to grammar that an individual requires for effective communication (e.g., pragmatic strategies and various linguistic forms, among others). Sociopragmatics is concerned with the social aspects related to the culture, as well as the interactive nature of communicative behavior. It is also related to social perceptions driving people’s interpretations. Social connections, observing proximity, the speaker’s and hearer’s rights, and requirements can be adjusted in communication (Taguchi, 2015). The domain of pragmatics covers various topics, such as politeness, illocutionary, proximity, interaction, movements, presupposition and entailment, and discourse, among others (Ishihara, 2010; Derakhshan, 2019).

### Explicit and implicit instructions

Norris and Ortega (2001) conducted a meta-analysis study on the types of L2 instruction, that revealed focused instruction to be more effective. In their study, categorized how pragmatic competence is taught into two groups: explicit vs. implicit treatments. It is claimed that explicit instruction, i.e., various classroom techniques employed to focus learners’ attention on structures and forms, is about to yield more positive outcomes than implicit instruction. The latter refers to methodological possibilities which enable students to infer rules unconsciously (Jeon and Kaya, 2006). Along with Norris and Ortega (2001), these two approaches are different in terms of the degrees of explanations provided in the class. In explicit instruction, learners are provided with rule explanations during instruction. As for interlanguage pragmatics, this has to do with the question of whether or not explicit metapragmatic information helps the learners to grasp and understand the target features more easily (Rose, 2005). Implicit instruction does not include any explanation of pragmatics or metapragmatic rule provision. The quasi-experimental studies have examined the extent to which these two modes of instruction are effective. To this end, they have compared an explicitly taught group with an implicitly taught group, with a control group sometimes included as well. The Implicit instruction of pragmatic competence has received much less attention than explicit pragmatic instruction. Along the same lines, Fukuya and Zhang (2002) refer to the fewer studies conducted on implicit pragmatic instruction, stating that this concern is understudied both conceptually and methodologically. Implicit instruction entails exposure to pragmatic input and providing no explicit explanation of the rules, i.e., meta-pragmatic information (Takahashi, 2001; Hernandez, 2011; Derakhshan and Shakki, 2020). In the same vein, many investigations have indicated the efficacy of explicit instruction, the significance of metapragmatic explanation, as well as the effect of the implicit intervention (Jeon and Kaya, 2006; Takahashi, 2010; Taguchi, 2015).

### Related studies on instruction

Quite a lot of research has been carried out on the possible role of instruction in the development of pragmatic competence, with the majority of them showing positive outcomes. The bulk of investigations conducted on the effect of L2 pragmatic instruction have mainly examined explicit instruction and implicit instruction about learning outcomes. To this end, they have compared explicit instruction with no explicit instruction (Kasper and Rose, 2002; Takahashi, 2010). Bacelar da Silva (2003) conducted a longitudinal study to examine the function of explicit teaching in the acquisition of polite refusal speech acts. Both sociopragmatic and pragmalinguistic aspects of refusal speech acts were taught by embedding various tasks with metapragmatic knowledge. Results indicated that explicit instruction enhances students’ pragmatic competence in refusal speech acts. In addition, Takahashi (2010) conducted a meta-analysis that involved the analysis of 49 studies. The results showed that explicit intervention was more effective than implicit intervention. The investigations covered in this analysis were all experimental, and they used a pre-test and post-test design. They concluded that overall, explicit interventions were found to be more effective in terms of the development of pragmatic features, particularly concerning some sociopragmatic features.

Moreover, Halenko and Jones (2011) conducted a study on 26 EFL learners in China. The results showed that explicit instruction enabled the participants to enhance their pragmatic abilities. This helped them to identify and produce pragmatically appropriate language forms. In their study, Nguyen et al. (2012) sought to examine the possible effect of explicit and implicit instruction on the acquisition of pragmatic competence. The sample consisted of sixty-nine Vietnamese students, who were split into three groups, namely, explicit, implicit, and control groups. The findings indicated that though the explicit instruction group performed better than the implicit one, students in both groups was more successful than the control group, thanks to the power of instruction. Similarly, Rajabi and Farahian (2013) carried out a study to assess the effect of instruction on the progress of pragmatic competence. The sample of the study was comprised of thirty-four Iranian EFL learners, who were assigned into two groups, i.e., an experimental and a control group. The former had awareness-raising instruction as their intervention. The findings showed that the group who received treatment outperformed the control group. Moreover, both explicit and implicit groups performed much better than the control group in terms of pragmatic performance.

Soler and Pitarch (2010) investigated the impact of teaching on raising pragmatic consciousness concerning the two phases involved in refusal speech acts, namely, planning and execution. Results revealed the efficacy of explicit instruction in channeling the learners’ attention to pragmatics. Results also showed that explicit instruction made a shift in learners’ attention as they concentrated on pragmalinguistic and sociopragmatic aspects rather than linguistic ones. In a review of more than 58 experimental studies in interlanguage pragmatics, Taguchi (2015) reviewed fifty-eight experimental studies on interlanguage pragmatics. The results showed that explicit instruction of pragmatic features was considered to be more operative compared to implicit instruction. The former focused on the form related to metapragmatic information. Input exposure proved to be inadequate in bringing about learning, even when the input had been highlighted by using enhancement techniques. Explicit explanations of the pragmalinguistic and sociopragmatic aspects of the speech act, along with raising awareness embedded in the tasks led to the learners’ metapragmatic awareness. Pragmatic features are more likely to lend themselves to explicit teaching than to implicit instruction (Rose, 2005).

In their review, Plonsky and Zhuang (2019) gathered a total of 50 research papers to examine the extent to which pragmatics instruction was effective. The results reinforced the previous meta-analyses acknowledging the effectiveness of explicit instruction compared to implicit instruction. They concluded that as pragmatics instruction provides ample opportunities for practice, it turned out to be more effective than instruction lacking practice, with longer instruction found to be better than the other one in general. Additionally, Doan (2019) scrutinized the efficiency of explicit and implicit instruction in terms of the learning outcomes regarding the acquisition of apology strategies among advanced L2 learners. The sample was made up of 30 potential participants who were divided into implicit and explicit instructions. The findings revealed that both training groups were far different in terms of their ability to produce speech acts after the treatment. Yet, explicit training was found to be advantageous to learners compared to the implicit training approach. Derakhshan and Shakki (2020) sought to consider the potential effect of the implicit and explicit instruction of metapragmatics on the Iranian EFL learners’ knowledge and their use of apology and refusal. The sample consisted of 49 EFL students, who were divided into three groups as follows: implicit instruction, explicit instruction, and control. The results showed an improvement in the learners’ pragmatic comprehension, with the explicit group outperforming the other groups.

## Conclusion, implications, and suggestions for further research

Great importance has been attached to the function of the instruction of L2 pragmatic competence in the language classroom. Research shows that there is a correlation between language proficiency and multiple components, including grammatical knowledge, syntax, morphology, phonology, and semantics; moreover, language proficiency is correlated with pragmatic competence, as well. Having no pragmatic competence would lead to miscommunications; consequently, there should be an emphasis on the incorporation of sociocultural awareness-raising modules in the classroom As stated by Soler (2001), a foreign language classroom is considered a good place where pragmatic competence can be taught and learned; The application of similar methodologies in EFL contexts enhances learners’ ability. Research on L2 pragmatics has revealed the positive outcomes of instruction compared to mere exposure to target pragmatic features regarding improving learners’ L2 pragmatic competence (Belz, 2007; Taguchi, 2015).

There are some of the contributions made by this study to the literature; for instance, instruction of pragmatics can help the students to acquire pragmatic competence; given that L2 education programs aimed at enabling the learners to use L2 appropriately and effectively in different interactional settings, so efforts should be made to raise learners’ pragmatic awareness. Moreover, learners should be equipped with some beneficial ways to engage in successful communication where there are different interlocutors; therefore, pragmatic competence needs to be an integral part of the L2 curriculum. The majority of investigations indicated that pragmatic instruction is more helpful than no instruction concerning the development of both linguistic and pragmatic competence (Tulgar, 2016). Given the results of the meta-analysis related to explicit instruction versus implicit instruction of pragmatics (Rose and Ng Kwai-Fun, 2001; Takahashi, 2001; Safont, 2005), it is concluded that explicit instruction yields better outcomes as this type of instruction involves the provision of metapragmatic information (e.g., rules of use and examples). The results revealed that EFL learners opted for explicit instruction as the language learners are rarely exposed to English in an EFL context like China. The effectiveness of explicit instruction may be attributable to its power in focusing learners’ attention on the target features. This enabled the learners to focus on the inputs containing it, providing them with more processing space for the exclusive processing of the target feature. In contrast, implicit teaching does not involve a direct focus on the feature in question (Roever, 2009). Provided that learners in the explicit instruction group performed much better on the post-test, it can be concluded that metapragmatic explanations helped learners to have linguistically correct and pragmatically proper statements.

Considering that pragmatic instruction makes an important contribution to L2 development, it is needed to incorporate the modules of pragmatic competence in combination with other language tasks to promote students’ consciousness regarding the proper use of the language. L2 input should be included along with other tasks in various contexts. This would make the learning process more meaningful. Elaborating on the importance of preparing and designing lessons, Solak and Bayar (2015) assert that the L2 curriculum must be organized based on a practice-based orientation rather than a conventional theory-based orientation. In these contexts, learners are provided with an opportunity to practice language, going beyond memorizing or mastering the linguistic forms (Eslami-Rasekh et al., 2004). Pragmatic knowledge and competence provide the learners with authentic input, enabling them to make informed pragmatic choices (opting for pragmatically suitable input in an EFL setting where they do not have access to outside-the-classroom opportunities for being exposed to pragmatic samples).

Given the important role of the appropriate use of language along with pragmatic competence in successful communications across cultures (Taguchi, 2015); L2 teachers are usually told to resort to explicit instruction of pragmatic features. They are also advised to present authentic models of L2 so that students can rehearse the appropriate use of language in a socially appropriate situation. Some recommendations have been made for teachers to focus L2 learners’ attention on both forms and functions through explicit instruction. Using various types of tasks in explicit instruction, teachers enable the L2 learners to work out the relationship between linguistic forms and functions. Examining the potential contribution made by explicit instruction to learning pragmatic features in an EFL context is of enormous importance in that EFL learners have very limited access to native speakers. Based on the research findings previously conducted, L2 teachers are advised to design tasks aimed at enhancing pragmatics embedded in explicit instruction (i.e., metapragmatic explanations). Explicit instruction allows the students to use the target forms, and to receive feedback from the teacher.

In addition, all L2 instructors must pave the way for students’ maximal exposure to the pragmatic features and students should practice these features in the socially appropriate situation. Moreover, L2 educators are required to have a good knowledge of L2 with an acceptable level of pragmatic awareness so that they can impart such knowledge to their learners in an effective manner. Teachers must have the prerequisite skills to teach these pragmatic aspects. To this effect, they must employ a diversity of tactics during their instruction. Therefore, EFL teachers are advised to raise L2 students’ attentiveness to the cultural aspects and serve as a facilitator. Accordingly, materials developers should use a diverse range of authentic appealing materials which encompasses the L2 pragmatic features and metapragmatic information. These features can be incorporated into both tasks and teaching materials.

Curriculum developers need to incorporate materials that explicitly contain various aspects of pragmatics. L2 practitioners also must be cognizant of the research evidence that the explicit instruction of pragmatics improves the EFL learners’ awareness, resulting in the build-up of pragmatic competence. This investigation gives some suggestions for the prospective research on pragmatic instruction in China, where such studies are scant. The current study contributes to the current literature by examining several approaches and putting forth some teaching models to prop up pragmatic competence teaching. Language stakeholders need to keep abreast of the latest research on pragmatics so that they can benefit from the latest implications in the instruction of pragmatics in their classes. Constructed on the demonstration of the efficacy of explicit instruction, further research should be done on the effect of methods employed to teach explicitly in this area (methods such as task-based learning or classic methods, among others). As a suggestion for further research, a longitudinal study can be conducted to examine the long-term role of explicit pragmatic instruction on the pace and quality of pragmatic competence. Based on the insights gained from such a study, teaching approaches can be modified accordingly. More empirical studies can be done in future to study the efficiency of other treatment types than explicit/implicit ones. It might be underlined that comprehending of pragmatic instruction can be exploited by scrutinizing the function of dichotomous interventional teaching methods. Regarding pedagogy, encouraging future paths to improve language learners’ pragmatic competence may be established in new areas and similarly in studying the impact of different teaching methods to the progress of proficiency with speech acts.

## Author contributions

The author confirms being the sole contributor of this work and has approved it for publication.
